# Studying the impact of marital status on diagnosis and survival prediction in pancreatic ductal carcinoma using machine learning methods

**DOI:** 10.1038/s41598-024-53145-6

**Published:** 2024-03-04

**Authors:** Qingquan Chen, Yiming Hu, Wen Lin, Zhimin Huang, Jiaxin Li, Haibin Lu, Rongrong Dai, Liuxia You

**Affiliations:** 1 The Second Affiliated Hospital of Fujian Medical University, Quanzhou, 362000 Fujian China; 2 The School of Public Health, Fujian Medical University, Fuzhou, 350108 Fujian China; 3 National Center for Chronic and Noncommunicable Disease Control and Prevention, Chinese Center for Disease Control and Prevention, Beijing, 100050 China; 4 Fuzong Clinical Medical College of Fujian Medical University, Fuzhou, 350108 Fujian China; 5 Anyang University, Anyang, 455000 China

**Keywords:** Cancer, Cancer epidemiology, Machine learning

## Abstract

Pancreatic cancer is a commonly occurring malignant tumor, with pancreatic ductal carcinoma (PDAC) accounting for approximately 95% of cases. According of its poor prognosis, identifying prognostic factors of pancreatic ductal carcinoma can provide physicians with a reliable theoretical foundation when predicting patient survival. This study aimed to analyze the impact of marital status on survival outcomes of PDAC patients using propensity score matching and machine learning. The goal was to develop a prognosis prediction model specific to married patients with PDAC. We extracted a total of 206,968 patient records of pancreatic cancer from the SEER database. To ensure the baseline characteristics of married and unmarried individuals were balanced, we used a 1:1 propensity matching score. We then conducted Kaplan–Meier analysis and Cox proportional-hazards regression to examine the impact of marital status on PDAC survival before and after matching. Additionally, we developed machine learning models to predict 5-year CSS and OS for married patients with PDAC specifically. In total, 24,044 PDAC patients were included in this study. After 1:1 propensity matching, 8043 married patients and 8,043 unmarried patients were successfully enrolled. Multivariate analysis and the Kaplan–Meier curves demonstrated that unmarried individuals had a poorer survival rate than their married counterparts. Among the algorithms tested, the random forest performed the best, with 0.734 5-year CSS and 0.795 5-year OS AUC. This study found a significant association between marital status and survival in PDAC patients. Married patients had the best prognosis, while widowed patients had the worst. The random forest is a reliable model for predicting survival in married patients with PDAC.

## Introduction

Pancreatic cancer is among the group of malignant tumors of the digestive tract that mainly originate from the ductal epithelium and alveolar cells of the pancreas. Approximately 95% of these tumors are pancreatic ductal carcinoma (PDAC), which has the fourth-highest mortality rate among cancers^1^. According to a global report in the year 2020, there were 495,773 newly diagnosed cases of pancreatic cancer and 466,003 fatalities. Notably, the incidence of pancreatic cancer was almost equivalent to the observed mortality rate^2^. Pancreatic cancer is projected to become the second most common cause of cancer-related deaths worldwide by 2030^3,4^. Despite significant advances in diagnosis and treatment, the prognosis of PDAC remains poor, with a 5-year survival rate of only 8–9%^5,6^. Surgical resection remains the only curative approach, but is often associated with poor outcomes and many postoperative complications^7^. Several factors, including age^8^, tumor stage, tumor size, lymph node metastasis, and treatment modalities, have been identified to impact the survival of PDAC patients. Notably, marital status has been shown to be an independent prognostic factor for perioperative and long-term survival among pancreatic cancer patients^9^. However, previous studies failed to consider the influence of confounding factors. Therefore, it is essential to account for confounding factors and thoroughly examine the association between marital status and the prognosis of PDAC in clinical practice.

Marriage, as a social phenomenon, represents a form of social support that bears great significance in the lives of human beings. Previous studies indicates that one's marital status can considerably influence their physical and mental well-being, including but not limited to cancer incidence and prognosis^10^. Marital support, emotional, financial stability and access to healthcare resources have been proposed to significantly influence cancer outcomes for married patients. In contrast, unmarried individuals are more likely to experience high levels of stress, social isolation, and lack of support, which may lead to poorer survival rates among cancer patients. Previous research has demonstrated that unmarried individuals have a higher risk of mortality in several types of cancers, including non-small cell lung cancer^11^, breast cancer^12^, laryngeal squamous cell carcinoma^13^, duodenal adenocarcinoma^14^, and esophageal cancer^15^, among others.

In this study, we utilized the SEER database to analyze the marital status of PDAC patients at the time of diagnosis and employed propensity score matching (PSM) to investigate the potential association between marital status and prognosis of PDAC. Additionally, we leveraged machine learning techniques to predict the survival time of married patients with PDAC.

## Materials and methods

### Ethics approval and consent to participate

The SEER database provides publicly available data for this study, which means that obtaining informed consent from participants or ethical approval from an institutional review board is not necessary. We obtained access to the 1979–2019 SEER Research Data File by signing a Data-Use Agreement that outlines the terms and conditions for access.

### Data source and patient selection

We utilized the SEER*Stat software (Version 8.4.0.1) to gather comprehensive data submitted to SEER until November 2021. In order to obtain patients with primary pancreatic site, we implemented the International Classification of Diseases for Oncology (ICD-O-3) topographical codes (C25.0-C25.3, C25.7-C25.9). We included patients diagnosed with ICD-O-3 histology/behavior codes of 8140/3 (adenocarcinoma) or 8500/3 (infiltrating duct adenocarcinoma) as part of our inclusion criteria. On the other hand, patients with pancreatic Islets of Langerhans (C25.4) tumor origin were excluded. Also, patients with missing/unknown/undifferentiated data on marital status, 6th AJCC stage and T/N/M stage, race, tumor differentiation, treatment information and the cause of death, as well as those with unknown or less than 1 month survival time were eliminated.

**Figure 1:**
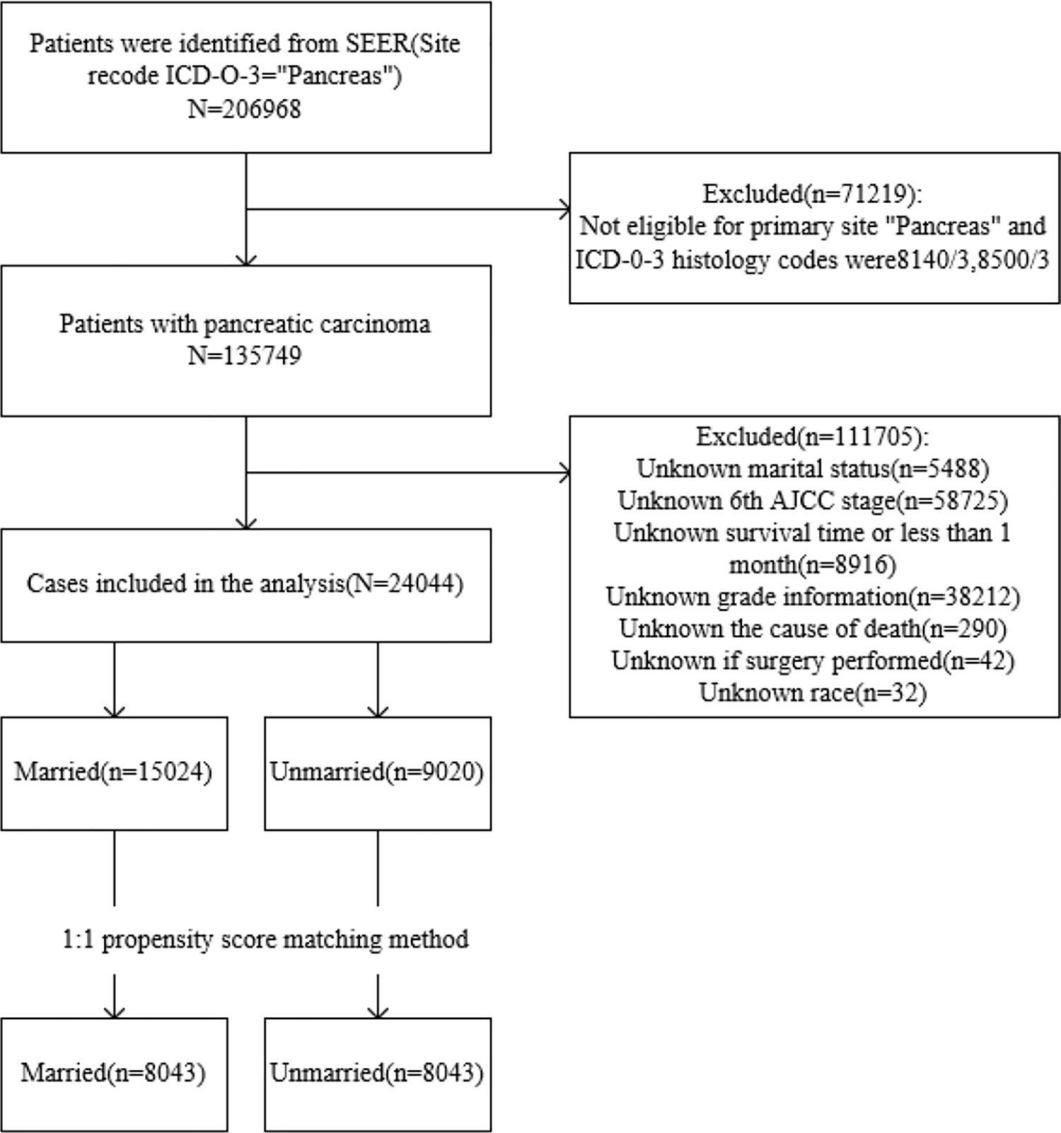
The flow-process diagram for selecting patients based on inclusion and exclusion criteria.

Following the application of our selection criteria, we identified a sum of 24,044 pancreatic ductal adenocarcinoma (PDAC) patients to serve as pertinent subjects for this investigation. Two patient clusters were then defined according to the marital status, namely, the married or unmarried group. A detailed flow-process diagram representation of our rigorous screening procedure is provided by Fig. 1.

### Variable classification

Our analysis incorporated a range of factors from the database such as sex, age at diagnosis, marital status, race, grade, TNM stage (6th), and primary site surgery. Age was dichotomized into two groups: those aged < 50 years and those aged ≥ 50 years. With regard to marital status, we distinguished participants as either married or unmarried groups based on their recorded statuses at the time of diagnosis. The unmarried group was composed of those who were divorced/separated, single, or widowed.

### Outcome measurement

In our study, we operationalized overall survival (OS) as the interval from the date of diagnosis to either the date of patient's decease or the last recorded follow-up instance if still alive. Similarly, cancer-specific survival (CSS) was gauged by determining the duration from the date of diagnosis to the date of death attributable solely to PDAC.

### Statistical analysis

To minimize potential confounding variables between married and unmarried patients, we gathered data on potential covariates such as sex, age, race, grade, TNM stage (6th), and primary site surgery for 1-to-1 propensity score matching (the nearest-neighbor method with a stringent caliper of 0.001), utilizing the R package of MatchIt. We utilized the chi-square test to assess differences in categorical variables and estimated OS and CSS by generating survival curves using the Kaplan–Meier method. Through the implementation of log-rank tests, we evaluated survival comparisons between distinct groups. To investigate possible prognostic factors and examine the hazard ratios, we employed both univariate and multivariable Cox proportional-hazards regression models.

With the aim of establishing a machine learning model, patients within the married group were partitioned into a training set and a test set at random, at an 8:2 ratio. Within the training set, we developed the K-nearest neighbor, artificial neural network, Naïve Bayes, and random forest models aimed at predicting the 5-year CSS and OS of married patients with PDAC. K-nearest neighbor (KNN) is a non-parametric algorithm that classifies or predicts outcomes based on the majority class or average of ‘k’ closest data points in the feature space. Artificial Neural Network (ANN) is composed of interconnected nodes (neurons) organized in layers, designed to learn and make predictions by adjusting the weights of connections during the training process. Naïve Bayes is a probabilistic algorithm that leverages Bayes' theorem, assuming independence between features, to calculate the likelihood of a particular class based on the observed data. Random Forest (RF) in machine learning prediction models is an ensemble learning method that constructs multiple decision trees during training and outputs the mode of the classes (classification) or the mean prediction (regression) of the individual trees for robust and accurate predictions.

All statistical analyses were carried out using R software (version 4.1.3) and SPSS software (version 25) with statistical significance set at two-sided *P* < 0.05.

## Results

### Pathological features and baseline characteristics

The SEER database provided data on a total of 206,968 PDAC patients for potential inclusion in our study. Ultimately, 24,044 individuals were deemed suitable after a series of screening procedures, as delineated in Fig. 1. Notably, of the eligible patients, 15,024 (62.49%) were classified as married and 9,020 (37.51%) as unmarried. Additional details regarding pathological features are elaborated in Table 1. Moreover, after executing the primary comparisons, significant differences were noted between the married and unmarried cohorts with regard to sex, race, TNM stage, and surgery status, with all values recorded as *P* ≤ 0.001 (Table 1).

**Table 1:** Baseline characteristics of patients patients with PDAC based on marital status.

Characteristic	Married (N = 15,024)	Unmarried (N = 9020)	*P*-value
Sex			
Male	8915 (59.3%)	3317 (36.8%)	< 0.001
Female	6109 (40.7%)	5703 (63.2%)	
Age at diagnosis			
< 50 years	925 (6.2%)	560 (6.2%)	0.894
≥ 50 years	14,099 (93.8%)	8460 (93.8%)	
Race			
White	12,579 (83.7%)	6948 (77.0%)	< 0.001
Black	1093 (7.3%)	1468 (16.3%)	
American Indian/Alaska native	64 (0.4%)	48 (0.5%)	
Asian or Pacific Islander	1288 (8.6%)	556 (6.2%)	
Grade			
Well differentiated	1602 (10.7%)	1046 (11.6%)	0.153
Moderately differentiated	7012 (46.7%)	4188 (46.4%)	
Poorly differentiated	6206 (41.3%)	3661 (40.6%)	
Undifferentiated	204 (1.4%)	125 (1.4%)	
TNM stage (6th)			
I	1147 (7.6%)	800 (8.9%)	< 0.001
II	7961 (53.0%)	4526 (50.2%)	
III	1420 (9.5%)	868 (9.6%)	
IV	4496 (29.9%)	2826 (31.3%)	
Surgery			
Yes	8401 (55.9%)	4403 (48.8%)	< 0.001
No	6623 (44.1%)	4617 (51.2%)	

### The primary comparison assessed the impact of marital status on OS and CSS

In univariate Cox regression analysis, mortality rates associated with PDAC were demonstrated to be significantly linked with seven variables, including sex, age, race, grade, TNM stage, primary site surgery, and marital status for both OS and CSS (*P* < 0.05; Table 2). Upon conducting multivariate Cox regression analysis to further investigate survival factors, we found that marital status, as well as sex, race, grade, TNM stage, and surgery status, emerged as independent prognostic factors that significantly influenced OS and CSS outcomes in patients with PDAC (*P* < 0.001; Table 3).

**Table 2:** Univariate analysis to assess the impact of marital status on OS/CSS in PDAC.

Variables	OS		CSS	
	HR (95% CI)	*P*-value	HR (95% CI)	*P*-value
Sex				
Male	Reference		Reference	
Female	0.952 (0.928–0.977)	< 0.001	0.958 (0.932–0.984)	0.002
Age at diagnosis				
< 50 years	Reference		Reference	
≥ 50 years	1.139 (1.078–1.203)	< 0.001	1.095 (1.036–1.158)	0.001
Race				
White	Reference		Reference	
Black	1.121 (1.074–1.170)	< 0.001	1.103 (1.055–1.153)	< 0.001
American Indian/Alaska native	1.213 (1.005–1.464)	0.045	1.252 (1.035–1.516)	0.021
Asian or Pacific Islander	0.966 (0.919–1.016)	0.178	0.980 (0.931–1.031)	0.432
Grade				
Well differentiated	Reference		Reference	
Moderately differentiated	1.130 (1.080–1.181)	< 0.001	1.145 (1.093–1.200)	< 0.001
Poorly differentiated	1.590 (1.520–1.664)	< 0.001	1.629 (1.554–1.707)	< 0.001
Undifferentiated	1.753 (1.558–1.972)	< 0.001	1.801 (1.586–2.034)	< 0.001
TNM stage (6th)				
I	Reference		Reference	
II	1.336 (1.267–1.408)	< 0.001	1.411 (1.333–1.493)	< 0.001
III	2.277 (2.134–2.430)	< 0.001	2.472 (2.308–2.648)	< 0.001
IV	4.003 (3.786–4.232)	< 0.001	4.391 (4.138–4.660)	< 0.001
Surgery				
No	Reference		Reference	
Yes	0.315 (0.306–0.324)	< 0.001	0.303 (0.294–0.312)	< 0.001
Marital status				
Unmarried	Reference		Reference	
Married	0.842 (0.820–0.865)	< 0.001	0.852 (0.829–0.876)	< 0.001

**Table 3:** Multivariate analysis to assess the impact of marital status on OS/CSS in PDAC.

Variables	OS		CSS	
	HR (95% CI)	*P*-value	HR (95% CI)	*P*-value
Sex				
Male	Reference		Reference	
Female	0.942 (0.917–0.967)	< 0.001	0.951 (0.925–0.978)	< 0.001
Age at diagnosis				
< 50 years	Reference		Reference	
≥ 50 years	1.241 (1.175–1.312)	< 0.001	1.197 (1.132–1.267)	< 0.001
Race				
White	Reference		Reference	
Black	1.039 (0.995–1.084)	0.085	1.022 (0.977–1.069)	0.348
American Indian/Alaska native	1.075 (0.890–1.297)	0.454	1.100 (0.909–1.332)	0.326
Asian or Pacific Islander	0.969 (0.921–1.018)	0.212	0.979 (0.930–1.031)	0.429
Grade				
Well differentiated	Reference		Reference	
Moderately differentiated	1.257 (1.202–1.315)	< 0.001	1.276 (1.218–1.338)	< 0.001
Poorly differentiated	1.585 (1.515–1.659)	< 0.001	1.618 (1.543–1.697)	< 0.001
Undifferentiated	1.494 (1.328–1.681)	< 0.001	1.520 (1.346–1.716)	< 0.001
TNM stage (6th)				
I	Reference		Reference	
II	1.432 (1.358–1.510)	< 0.001	1.518 (1.434–1.607)	< 0.001
III	1.447 (1.353–1.548)	< 0.001	1.551 (1.445–1.665)	< 0.001
IV	2.206 (2.079–2.341)	< 0.001	2.380 (2.235–2.536)	< 0.001
Surgery				
No	Reference		Reference	
Yes	0.392 (0.378–0.407)	< 0.001	0.382 (0.367–0.397)	< 0.001
Marital status				
Unmarried	Reference		Reference	
Married	0.840 (0.817–0.864)	< 0.001	0.851 (0.826–0.876)	< 0.001

### The secondary comparison assessed the impact of marital status on both OS and CSS

**Figure 2:**
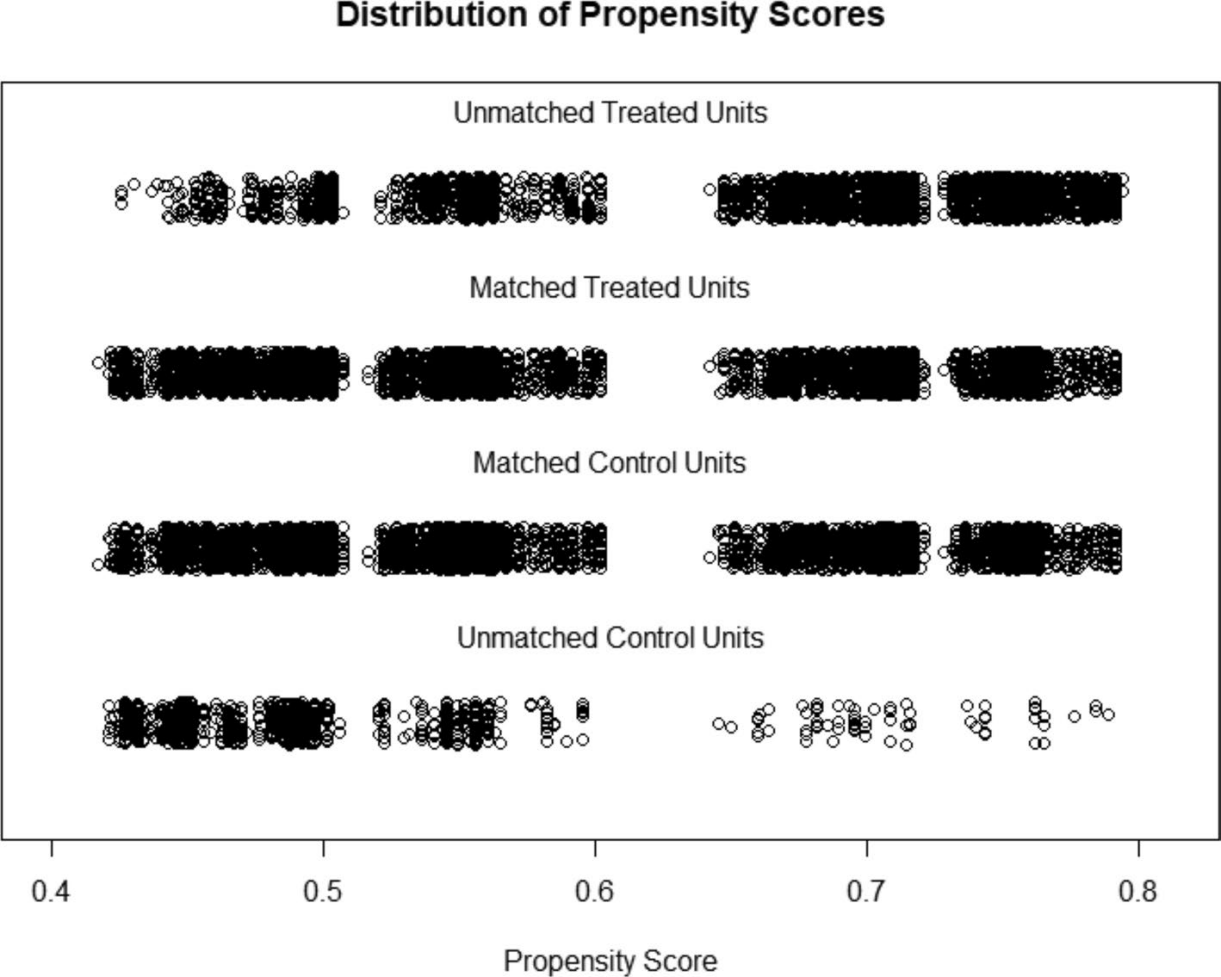
Propensity score matching for married and unmarried groups.

To eliminate for potential confounding variables such as age, sex, and race between the married and unmarried groups, we employed the 1:1 propensity score matching method. After matching, 8043 married patients and an equal number of unmarried patients (for a total of 8043 individuals) were successfully enrolled. Notably, the baseline characteristics were found to be well-balanced between the two groups (Table 4; Fig. 2), and no significant differences were observed (*P* > 0.05).

**Table 4:** Baseline characteristics of patients patients with PDAC based on marital status after propensity-score matching.

Characteristic	Married (N = 8043)	Unmarried (N = 8043)	*P*-value
Sex			
Male	3248 (40.4%)	3248 (40.4%)	1
Female	4795 (59.6%)	4795 (59.6%)	
Age at diagnosis			
< 50 years	470 (5.8%)	506 (6.3%)	0.248
≥ 50 years	7573 (94.2%)	7537 (93.7%)	
Race			
White	6570 (81.7%)	6570 (81.7%)	1
Black	916 (11.4%)	916 (11.4%)	
American Indian/Alaska native	25 (0.3%)	25 (0.3%)	
Asian or Pacific Islander	532 (6.6%)	532 (6.6%)	
Grade			
Well differentiated	893 (11.1%)	885 (11.0%)	0.997
Moderately differentiated	3723 (46.3%)	3724 (46.3%)	
Poorly differentiated	3333 (41.4%)	3341 (41.5%)	
Undifferentiated	94 (1.2%)	93 (1.2%)	
TNM stage (6th)			
I	620 (7.7%)	620 (7.7%)	0.998
II	4148 (51.6%)	4148 (51.6%)	
III	753 (9.4%)	760 (9.4%)	
IV	2522 (31.4%)	2515 (31.3%)	
Surgery			
Yes	3869 (48.1%)	3869 (48.1%)	1
No	4174 (51.9%)	4174 (51.9%)

The findings indicate that, with the exception of race, all baseline characteristics were significant predictors of both OS and CSS (Table 5). In the univariate analysis after propensity-score matching, being unmarried (with reference to married) remained a statistically significant predictive risk factor of death (OS: HR = 0.870, 95% CI = 0.842–0.898, *P* < 0.001; CSS: HR = 0.882, 95% CI = 0.853–0.912, *P* < 0.001). Upon subjecting relevant variables to further multivariate analysis, all components maintained independent significance in predicting OS/CSS with the exception of sex. Moreover, unmarried status (with reference to married) exhibited a noteworthy negative influence on survival outcomes (OS: HR = 0.834, 95% CI 0.808–0.861, *P* < 0.001; CSS: HR = 0.845, 95% CI 0.817–0.873, *P* < 0.001; Table 5). It is worth noting that patients diagnosed prior to age 50, those with stage I cancer, well-differentiated tumors, and those who had undergone surgery were observed to be more likely to experience an improvement in both OS and CSS compared to their respective reference groups (Table 5).

**Table 5:** Univariate and multivariate analysis of the impact of marital status on survival outcomes in PDAC.

Variables	OS				CSS			
	Univariate analysis		Multivariate analysis		Univariate analysis		Multivariate analysis	
	HR (95% CI)	*P*-value	HR (95% CI)	*P*-value	HR (95% CI)	*P*-value	HR (95% CI)	*P*-value
Sex								
Male	Reference		Reference		Reference		Reference	
Female	0.924 (0.894–0.955	< 0.001	0.973 (0.942–1.006)	0.103	0.927 (0.897–0.959)	< 0.001	0.980 (0.947–1.014)	0.241
Age at diagnosis								
< 50 years	Reference		Reference		Reference		Reference	
≥ 50 years	1.130 (1.055–1.209)	< 0.001	1.248 (1.166–1.337)	< 0.001	1.086 (1.014–1.164)	0.019	1.202 (1.121–1.289)	< 0.001
Race								
White	Reference		Reference		Reference		Reference	
Black	1.090 (1.036–1.146)	0.001	1.043 (0.991–1.097)	0.105	1.081 (1.026–1.140)	0.004	1.033 (0.980–1.089)	0.232
American Indian/Alaska native	1.031 (0.777–1.369)	0.833	0.968 (0.729–1.286)	0.823	1.052 (0.787–1.405)	0.732	0.979 (0.732–1.308)	0.884
Asian or Pacific Islander	0.998 (0.935–1.065)	0.953	0.948 (0.888–1.012)	0.107	1.020 (0.954–1.090)	0.560	0.965 (0.902–1.031)	0.292
Grade								
Well differentiated	Reference		Reference		Reference		Reference	
Moderately differentiated	1.094 (1.036–1.155)	0.001	1.232 (1.167–1.302)	< 0.001	1.108 (1.047–1.173)	< 0.001	1.254 (1.184–1.328)	< 0.001
Poorly differentiated	1.545 (1.462–1.632)	< 0.001	1.542 (1.459–1.630)	< 0.001	1.589 (1.501–1.683)	< 0.001	1.584 (1.496–1.678)	< 0.001
Undifferentiated	1.666 (1.428–1.944)	< 0.001	1.379 (1.181–1.609)	< 0.001	1.699 (1.449–1.992)	< 0.001	1.397 (1.191–1.639)	< 0.001
TNM stage (6th)								
I	Reference		Reference		Reference		Reference	
II	1.321 (1.237–1.410)	< 0.001	1.418 (1.327–1.514)	< 0.001	1.403 (1.307–1.506)	< 0.001	1.511 (1.408–1.623)	< 0.001
III	2.331 (2.150–2.527)	< 0.001	1.415 (1.301–1.539)	< 0.001	2.549 (2.340–2.776)	< 0.001	1.520 (1.391–1.662)	< 0.001
IV	4.054 (3.783–4.344)	< 0.001	2.193 (2.037–2.362)	< 0.001	4.473 (4.154–4.816)	< 0.001	2.371 (2.191–2.565)	< 0.001
Surgery								
No	Reference		Reference		Reference		Reference	
Yes	0.312 (0.302–0.323)	< 0.001	0.387 (0.369–0.406)	< 0.001	0.299 (0.288–0.310)	< 0.001	0.375 (0.357–0.394)	< 0.001
Marital status								
Unmarried	Reference		Reference		Reference		Reference	
Married	0.870 (0.842–0.898)	< 0.001	0.834 (0.808–0.861)	< 0.001	0.882 (0.853–0.912)	< 0.001	0.845 (0.817–0.873)	< 0.001

**Figure 3:**
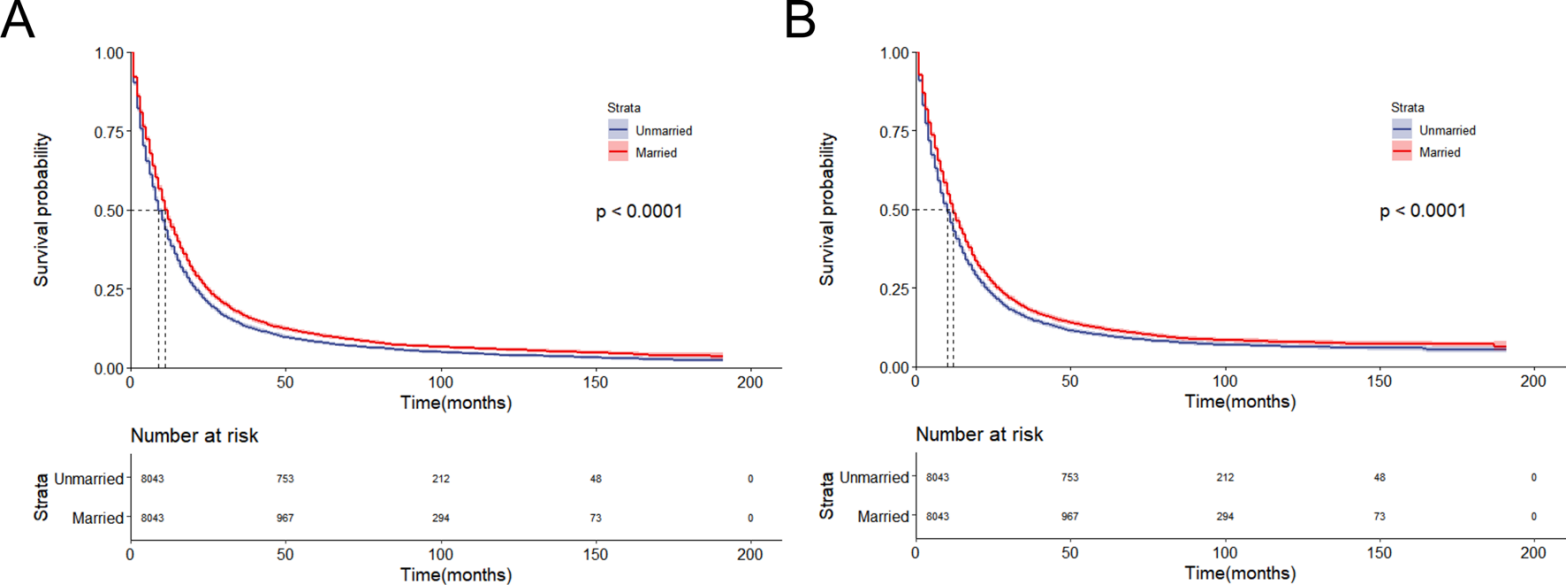
Kaplan–Meier survival curves of PDAC patients between married and unmarried groups. (**A)** Overall survival. (**B)** Cancer-specific survival.

**Figure 4:**
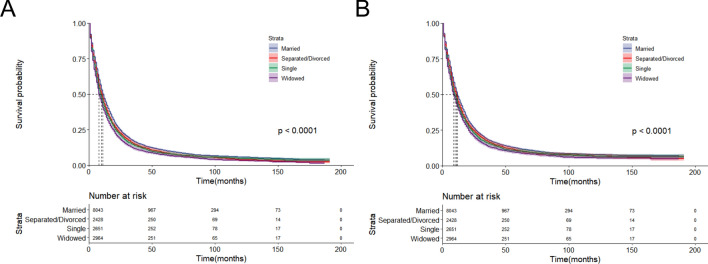
Kaplan–Meier survival curves of PDAC patients among married, separated/divorced, single and widowed. (**A)** Overall survival. (**B)** Cancer-specific survival.

The Kaplan–Meier curves presented in Fig. 3 indicate that unmarried individuals have a significantly lower survival rate than married individuals (*P* < 0.001). To further investigate the prognosis of different unmarried statuses, we grouped unmarried patients into separated/divorced, single, and widowed subgroups. As shown in Fig. 4, we found that there was a significant difference between their OS/CSS and different marital statuses (*P* < 0.001).

**Figure 5:**
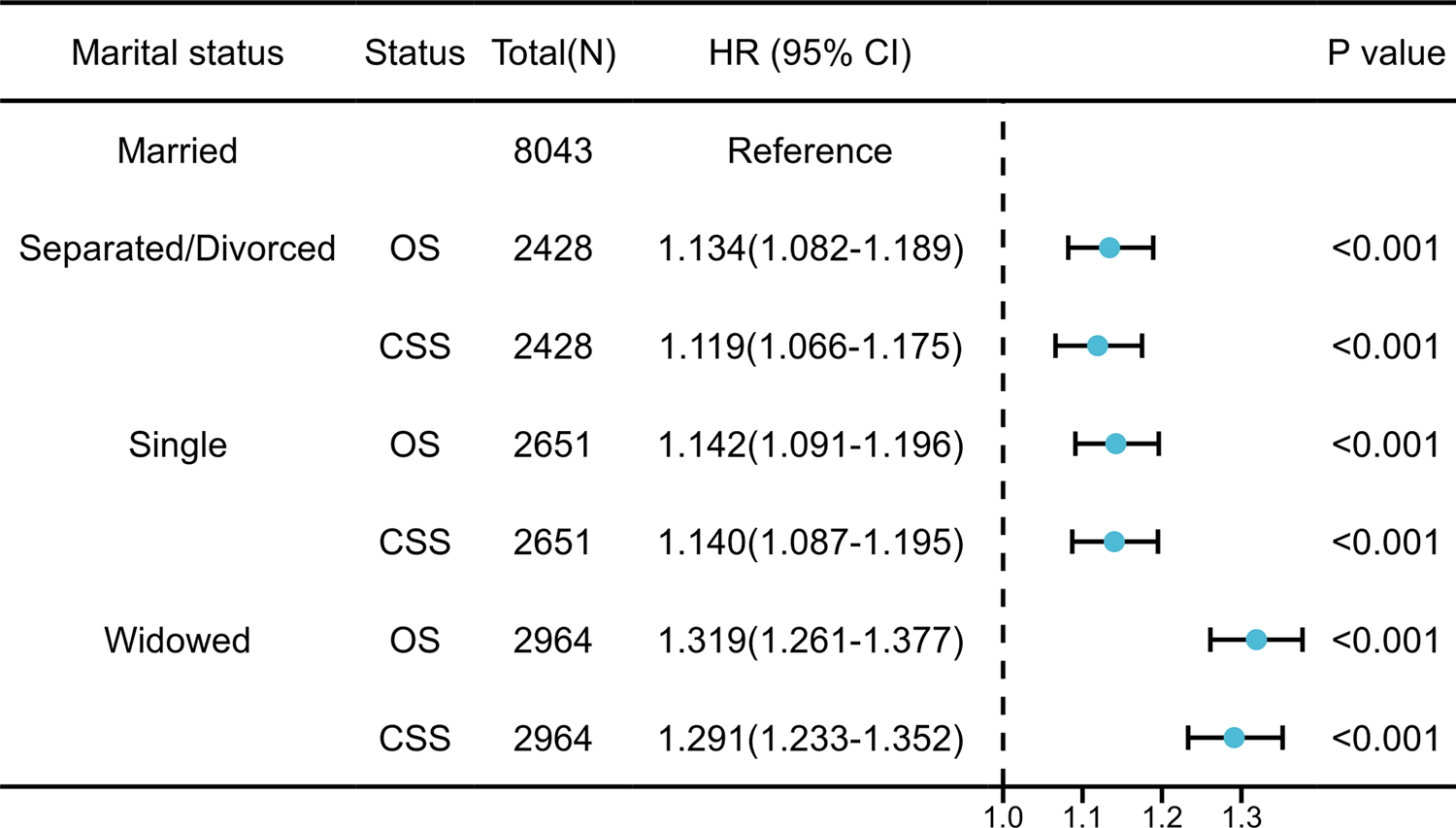
The impact of marital status on CSS and OS in the secondary comparison. Circles represent the aHRs with the 95% CIs indicated by horizontal bars.

In the secondary comparison, we utilized a forest plot to evaluate the impact of different kinds of unmarried statuses versus married status. As illustrated in Fig. 5, separated/divorced patients (OS: aHR = 1.134, 95% CI 1.082–1.189, *P* < 0.001; CSS: aHR = 1.119, 95% CI 1.066–1.175, *P* < 0.001), single patients (OS: aHR = 1.142, 95% CI 1.091–1.196, *P* < 0.001; CSS: aHR = 1.140, 95% CI 1.087–1.195, *P* < 0.001), and widowed patients (OS: aHR = 1.319, 95% CI = 1.261–1.377, *P* < 0.001; CSS: aHR = 1.291, 95% CI 1.233–1.352, *P* < 0.001) exhibit poorer survival outcomes relative to married patients. Additionally, we observed that widowed patients have the highest risk of death among the three unmarried statuses (Figs. 4 and 5).

### Machine-learning based outcome prediction in patients who married

**Figure 6:**
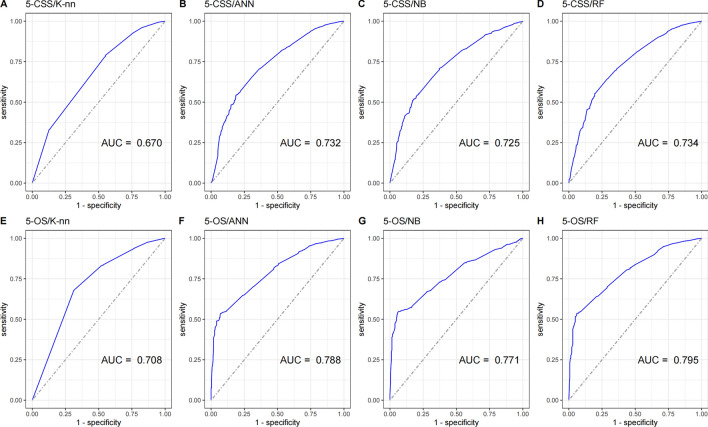
Comparison of receiver operating characteristics (ROC) curves between the four machine learning models for 5-year CSS and 5-year OS prediction.

To explore the factors that influence the survival of married patients with PDAC, we utilized age, sex, race, tumor differentiation, TNM stage, and surgery status as input parameters for developing machine learning prediction models of the 5-year CSS and 5-year OS. The performance metrics of the algorithms for the four models are presented in Table 6. Among the machine learning models, the random forest model exhibits superior discrimination performance. For predicting the 5-year CSS, the random forest model achieves an AUROC of 0.734, accuracy of 0.592, recall of 0.552, specificity of 0.806, precision of 0.939, and F1 score of 0.695. The 5-year OS results are 0.795, 0.572, 0.536, 0.940, 0.989, and 0.695 for AUROC, accuracy, recall, specificity, precision, and F1 score, respectively. Artificial neural network, naïve bayes, and k-nearest neighbor follow with AUROCs of 0.788, 0.771, and 0.708, respectively. Receiver operating characteristics (ROC) curves and AUROCs of the four models are displayed in Fig. 6. By using GridSearch, the hyperparameters of the optimal random forest model were: N_estimators = 100, Max_depth = 10, Min_samples_leaf = 2, Min_samples_split = 4, Max_features = auto (Table S1).

**Table 6:** Discrimination tests of four machine learning models for predicting 5-year CSS and 5-year OS.

Algorithm	Discrimination tests					
	AUROC (95% CI)	Accuracy (95% CI)	Recall (95% CI)	Specificity (95% CI)	Precision (95% CI)	F1-score (95% CI)
5-CSS						
K-nearest neighbor	0.670 (0.644–0.695)	0.736 (0.736–0.736)	0.789 (0.774–0.805)	0.449 (0.404–0.494)	0.886 (0.873–0.899)	0.835 (0.821–0.849)
Artificial neural network	0.732 (0.707–0.756)	0.586 (0.586–0.586)	0.544 (0.525–0.564)	0.812 (0.777–0.847)	0.940 (0.928–0.952)	0.689 (0.671–0.708)
Naïve Bayes	0.725 (0.701–0.749)	0.566 (0.566–0.566)	0.519 (0.499–0.538)	0.821 (0.786–0.855)	0.940 (0.928–0.952)	0.669 (0.649–0.687)
Random forest	**0.734 (0.709–0.758)**	0.592 (0.591–0.592)	0.552 (0.533–0.571)	0.806 (0.77–0.841)	0.939 (0.927–0.951)	0.695 (0.677–0.714)
5-OS						
K-nearest neighbor	0.708 (0.676–0.74)	0.678 (0.677–0.678)	0.676 (0.659–0.694)	0.689 (0.634–0.745)	0.957 (0.948–0.966)	0.792 (0.778–0.808)
Artificial neural network	0.788 (0.764–0.812)	0.570 (0.57–0.571)	0.535 (0.517–0.554)	0.929 (0.898–0.96)	0.987 (0.981–0.993)	0.694 (0.677–0.711)
Naïve Bayes	0.771 (0.748–0.794)	0.579 (0.579–0.579)	0.544 (0.525–0.562)	0.940 (0.912–0.969)	0.989 (0.984–0.995)	0.702 (0.685–0.718)
Random forest	**0.795 (0.771–0.818)**	0.572 (0.572–0.572)	0.536 (0.517–0.555)	0.940 (0.912–0.969)	0.989 (0.984–0.994)	0.695 (0.678–0.712)

Significant values are in bold.

**Figure 7:**
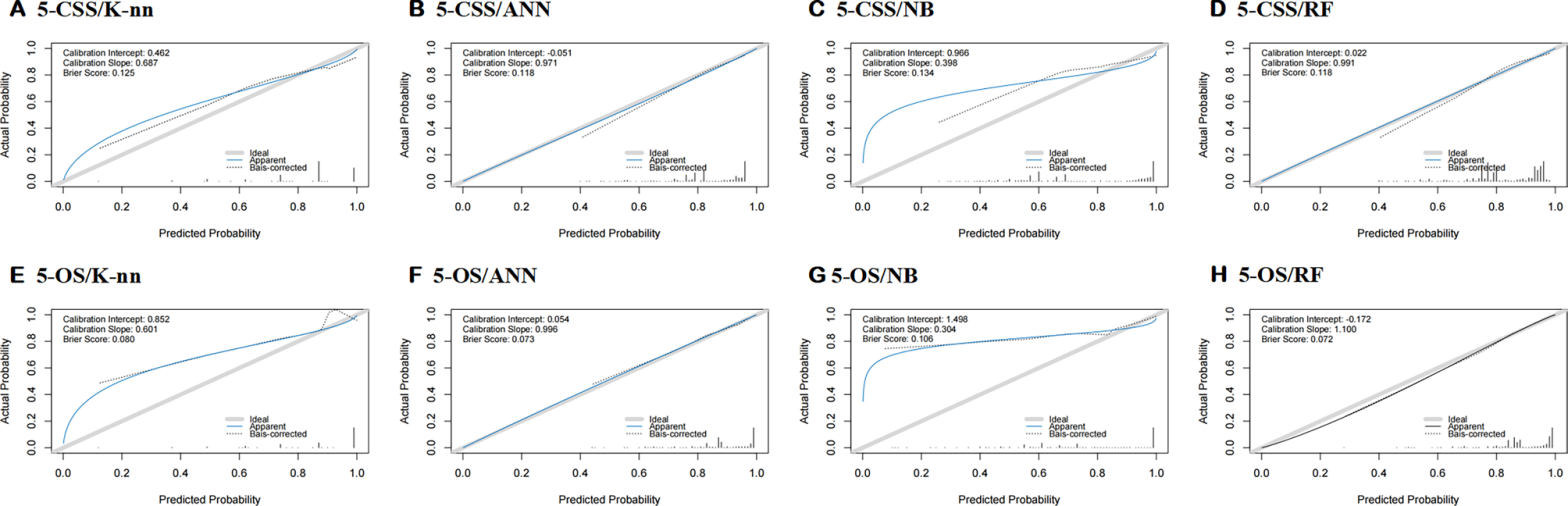
Calibration curves for testing the stability of four prediction models. The logical calibration curve is shown in solid blue, and the statistics are displayed in the top left corner of each graph.

The calibration curves demonstrated an excellent agreement between predictions and observations (Fig. 7). For predicting the 5-year CSS, the k-nearest neighbor, artificial neural network, naïve bayes and random forest models gave brier scores of 0.125, 0.118, 0.134, and 0.118, respectively. Similarly, while the 5-year OS, brier scores of 0.080, 0.073, 0.106, and 0.072 were obtained using the same models, as outlined in Table 7.

**Table 7:** Calibration tests of four machine learning models for predicting 5-year CSS and 5-year OS.

Algorithm	Calibration		
	Brier score	Slope	Intercept
5-CSS			
K-nearest neighbor	0.125	0.687	0.462
Artificial neural network	0.118	0.971	− 0.051
Naïve Bayes	0.134	0.398	0.966
Random forest	0.118	0.991	0.022
5-OS			
K-nearest neighbor	0.080	0.601	0.852
Artificial neural network	0.073	0.996	0.054
Naïve Bayes	0.106	0.304	1.498
Random forest	0.072	1.100	− 0.172

**Figure 8:**
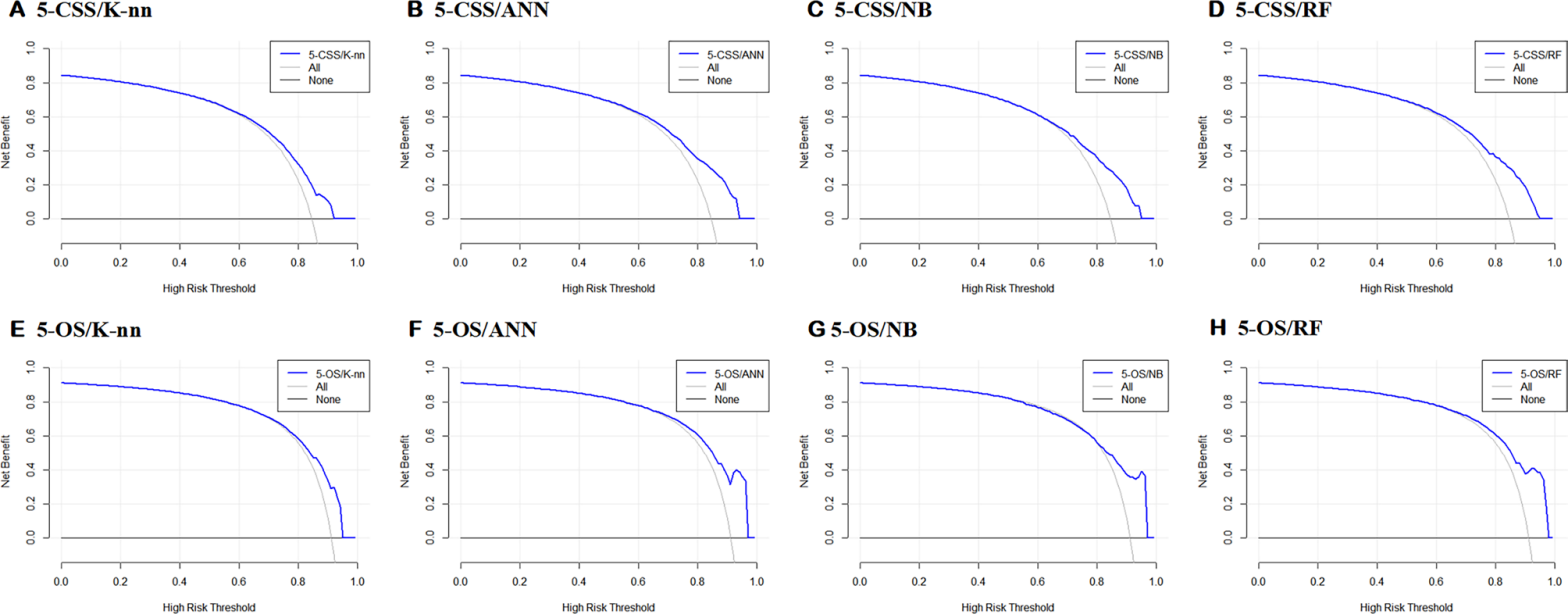
Decision curve analysis of eight prediction models.

**Figure 9:**
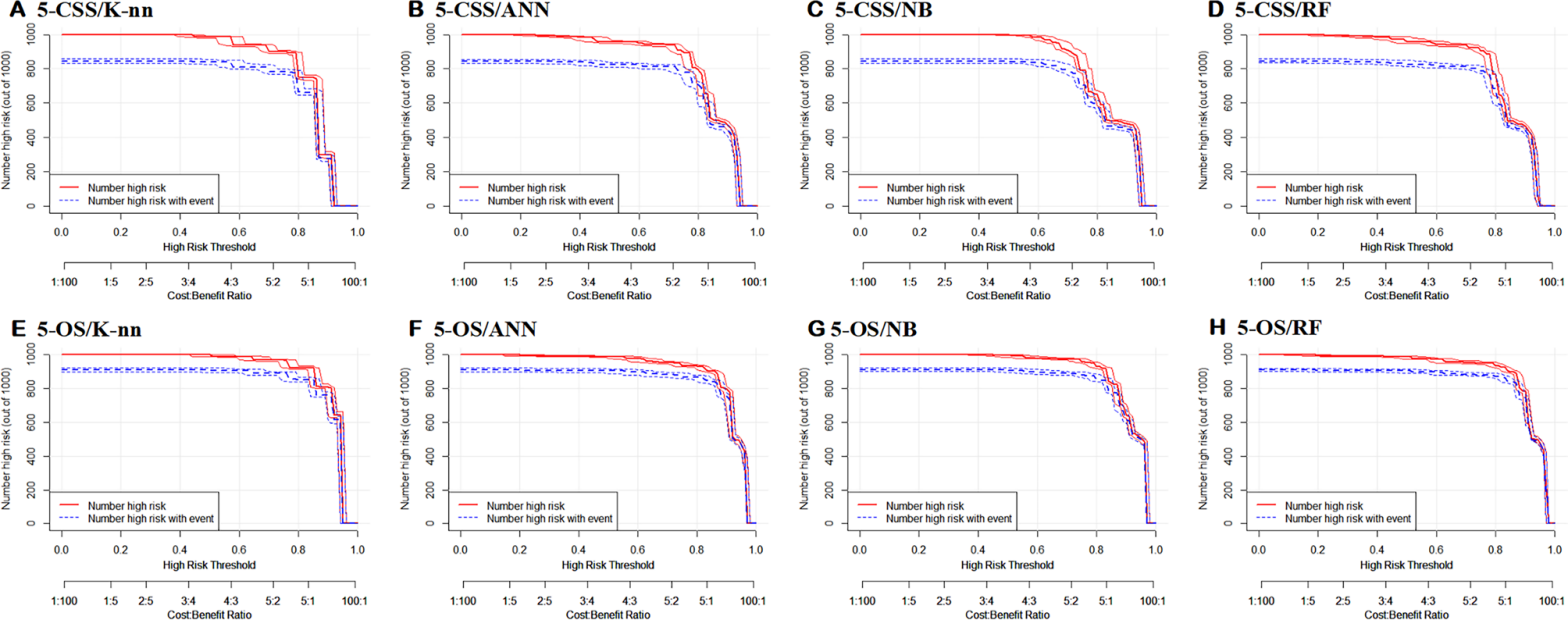
Clinical impact curve analysis of eight prediction models.

In this study, the clinical effectiveness of four predictive models was assessed using decision curves and clinical impact curves. The DCA curve (Fig. 8) indicated that the random forest model had a greater net benefit compared to the "treat none" or "treat all" schemes across a threshold probability range of 0.6 to 1.0. Further, the random forest model exhibited superior clinical impact when compared to the other models. Notably, when the threshold probability was set above 75% (Fig. 9), the number of positive cases predicted by the models (i.e., those at high risk) was closely matched the number of true-positive cases (i.e., those who actually had high-risk outcomes). Considering all four evaluation metrics, it can be concluded that the random forest algorithm performed the best for prediction purposes and could offer more precise and systematic treatment guidance and support to married patients with PDAC.

## Discussion

Marital status has been shown to be associated with survival in chronic diseases such as cancer, with married individuals having a longer life expectancy and better quality of life in various diseases. For instance, Cheng Xu et al. used a matching method to discover that married patients had better 5-year CSS/OS than unmarried patients with NPC from 1973 to 2012^16^. Gino Inverso et al. also observed a significant protective impact of marital status on metastatic oral cancer and laryngeal cancer^17^. However, while studies have confirmed that marital status is a prognostic factor for pancreatic cancer using SEER database, the available studies have failed to exclude confounding factors^9,18,19^. Previous studies have found that sex, age, stage, race^20^ and surgery^21^ were associated with the survival of PDAC patients. Therefore, to improve comparability between married and unmarried patients, we conducted a 1:1 propensity matching using the SEER database to screen eligible patients with PDAC, resulting in relatively reliable results based on well-matched datasets. As well as a larger sample size, our study could provide more robust results compared with previous studies. Male sex, age over 50 years, higher TNM stage, worse tumor differentiation, and no surgical treatment were determined to be risk factors for prognosis, and married patients had better survival outcomes; however, their unmarried counterparts had significantly poor OS/CSS.

The current study found that marital status plays a significant role in PDAC patients, and we suspect that several possible reasons exist. First, marriage provides positive social support. It has been found that widowed, divorced, and separated individuals lack legal relationships, partner support and help during diagnosis and treatment, and are hence at a higher risk of psychological distress^22^. Similarly, relative to married patients, unmarried patients are more likely to experience negative emotional states for a prolonged period due to the absence of social support and partner companionship, which could lead to physiological dysfunctions resulting from long-term exposure to glucocorticoids and catecholamines, negatively affecting the tumor microenvironment and tumor growth, migration and stimulating angiogenesis, thus affecting the prognosis^23^. Healthy marital status plays an essential role in establishing a good psychological state, reducing negative emotions, such as anxiety and depression, and improving survival rate^10,24^. Secondly, stable marital relationships are typically associated with higher economic status, and family members such as spouses and children may provide financial and spiritual support for long-term treatment^25^. In other words, stable marital status can improve patient compliance with the treatment regimen comparatively^26^. In addition, married people with a good economic base are more likely to purchase health insurance and can receive some Medicaid at the time of diagnosis^27^. Furthermore, studies have found that patients with private health insurance are found in a greater proportion of early stages of cancer, have longer survival time and better prognosis. Patients without private health insurance, on the other hand, are usually detected at an advanced stage of cancer and have a poor prognosis^28^. Thirdly, married individuals typically adopt healthier lifestyles, with better diets, more exercise, and less substance abuse, contributing to better healthy outcomes. It has been shown that bad habits such as smoking and alcoholism are risk factors for the development of pancreatic ductal carcinoma, while unmarried people are more likely to be infected^26^. Lastly, in daily life, family members of married patients are more likely to detect early symptoms of PDAC, resulting in early detection and diagnosis and positive impacts on disease treatment.

Recently, machine learning has been widely used in the medical field^29^. Some researchers have developed prognostic prediction models for pancreatic cancer using machine learning methods, because machine learning algorithms are more accurate than traditional statistical methods in predicting survival outcome in the fifth year^30^. Specifically, in this study, the k-nearest neighbor, artificial neural network, naïve bayes, and random forest algorithms were used to predict the 5-year CSS and OS for married patients with PDAC. The results indicate that the random forest algorithm outperformed the other models in predicting 5-year CSS/OS, especially in its good discriminative performance and its AUROC value was high, indicating that the model could better distinguish between lives and deaths. Furthermore, since random forest has good generalization capability, it can avoid the overfitting issue. Moreover, the random forest model stands out in prognostic prediction tasks due to its superior predictive accuracy, robustness to noisy data, interpretability through feature importance analysis, capacity to handle non-linearity, generalization to unseen data, stability, and ease of implementation. Our study presents the first predictive model based on machine learning algorithms that predicts the survival impact of married patients with PDAC, which demonstrates excellent performance and provides doctors with an easily accessible and more accurate survival prediction tool for married patients with PDAC, which may guide clinical practice better. It must be admitted that with the rapid evolution of machine learning, particularly in deep learning, ensemble methods, and reinforcement learning, have led to models with increased predictive power. Therefore, we believe that our prediction model would be improved with the development of machine learning and provide more accurate prediction.

Although marriage has positive impacts on cancer outcomes, it is vital to note that not all marriages are beneficial for health. Marital conflict and stress may lead to negative effects on health, including increased risk of depression, anxiety, and heart disease. Thus, future studies should investigate the quality of marital relationships and the impact of marital therapy on cancer patient health outcomes.

## Limitations

Although the results of this study indicates that marital status is a significant prognostic factor for PDAC, it is not without limitations.

Firstly, the SEER database contains limited clinical characteristics of patients and lacks critical risk factors such as tobacco smoking, alcohol consumption, type 2 diabetes, chronic pancreatitis, and family history of pancreatic cancer^31^. The lack of accurate screening before data matching may result in biased conclusions. Particularly, diabetes confers a 3.05-fold increased risk of PDAC onset in diabetic individuals compared to non-diabetic individuals^32^. Secondly, the absence of data on the quality of life of patients in the SEER database, such as socioeconomic level and living environment, the quality of life of patients were not available for inclusion in our analysis. Thirdly, the marital status extracted in this study was recorded only at diagnosis, and dynamic follow-up surveys assessing changes in marital status during PDAC treatment were not taken. This may pose information bias on clinical outcomes. We cannot understand marital status during the later treatment of patients, which may have some information bias. Fourthly, our classification of PDAC patients living together with partners but not legally married as single patients may underestimate survivorship outcomes among this group, which may be better than that of unmarried or single patients. Despite the limited proportion of such patients, they may impact the conclusions of this study. Finally, this study's generalizability may be limited to the population under investigation, as cultural variations, disparities in living standards, and economic differences between countries may influence the applicability of these findings to patients in other regions.

## Conclusion

Our study provides evidence that marital status is an independent prognostic factor for PDAC. Future studies should investigate the mechanisms behind this association and the impact of marital quality and therapy on cancer outcomes. We established machine learning predictions about the survival of married patients with PDAC, with the RF model performing best.

### Supplementary Information

Supplementary Table S1.

## Data Availability

The data used in this study are available free of charge online at http://www.seer.cancer.gov on request.

## Abbreviations

aHR: Adjusted hazard ratio
ANN: Artificial neural network
CI: Confidence interval
CSS: Cause-specific survival
K-nn: K-nearest neighbor
NB: Naïve Bayes
RF: Random forest
ROC: Receiver operating characteristics
OS: Overall survival
PDAC: Pancreatic ductal carcinoma
