# Author Correction: Studying the impact of marital status on diagnosis and survival prediction in pancreatic ductal carcinoma using machine learning methods

**DOI:** 10.1038/s41598-024-57179-8

**Published:** 2024-03-22

**Authors:** Qingquan Chen, Yiming Hu, Wen Lin, Zhimin Huang, Jiaxin Li, Haibin Lu, Rongrong Dai, Liuxia You

**Affiliations:** 1 The Second Affiliated Hospital of Fujian Medical University, Quanzhou, 362000 Fujian China; 2 The School of Public Health, Fujian Medical University, Fuzhou, 350108 Fujian China; 3 National Center for Chronic and Noncommunicable Disease Control and Prevention, Chinese Center for Disease Control and Prevention, Beijing, 100050 China; 4 Fuzong Clinical Medical College of Fujian Medical University, Fuzhou, 350108 Fujian China; 5 Anyang University, Anyang, 455000 China

Correction to: *Scientific Reports* 10.1038/s41598-024-53145-6, published online 04 March 2024

In the original version of this Article, the Affiliations were not listed in the correct order. The correct affiliations are listed below:

Affiliation 1

The Second Affiliated Hospital of Fujian Medical University, Quanzhou 362000, Fujian, China.

Affiliation 2

The School of Public Health, Fujian Medical University, Fuzhou 350108, Fujian, China.

Affiliation 3

National Center for Chronic and Noncommunicable Disease Control and Prevention, Chinese Center for Disease Control and Prevention, Beijing 100050, China.

Affiliation 4

Fuzong Clinical Medical College of Fujian Medical University, Fuzhou 350108, Fujian.

Affiliation 5

Anyang University, Anyang 455000, China.

The original Article has been corrected.

