# Supplementary material for: Studying the impact of marital status on diagnosis and survival prediction in pancreatic ductal carcinoma using machine learning methods
Source: Sci Rep. 2024 Mar 4;14:5273. doi: 10.1038/s41598-024-53145-6 (PMC10912082; doi:10.1038/s41598-024-53145-6)
Supplement: Supplementary file 1 — Supplementary Table S1. [file 41598_2024_53145_MOESM1_ESM.docx]

**Table S1. Optimum parameter combination using GridSearch.**

| **Model** | **Hyperparameters** | **Optimal value** |
| --- | --- | --- |
| 5-CSS | | |
| K-nearest neighbor | N_neighbors | 8 |
|  | Weights | uniform |
|  | Algorithm | auto |
|  | Leaf_size | 30 |
|  | Metric | minkowski |
|  | p | 2 |
|  | Metric_params | None |
|  | N_jobs | 1 |
| Artificial neural network | Hidden_layer_sizes | (5, 5) |
|  | Activation | logistic |
|  | Solver | adam |
|  | Alpha | 0.0001 |
|  | Batch_size | auto |
|  | Learning_rate | 0.01 |
|  | Power_t | 0.5 |
|  | Max_iter | 1000 |
|  | Random_state | RandomState |
|  | Shuffle | True |
|  | Tol | 1.0E-4 |
|  | Learning_rate_int | 0.001 |
| Naïve Bayes | Alpha | 1 |
|  | Binarize | 0 |
|  | Fit_prior | True |
| Random forest | N_estimators | 100 |
|  | Max_depth | 10 |
|  | Min_samples_leaf | 2 |
|  | Min_samples_split | 4 |
|  | Max_features | auto |
| 5-OS | | |
| K-nearest neighbor | N_neighbors | 5 |
|  | Weights | uniform |
|  | Algorithm | auto |
|  | Leaf_size | 30 |
|  | Metric | minkowski |
|  | p | 2 |
|  | Metric_params | None |
|  | N_jobs | 1 |
| Artificial neural network | Hidden_layer_sizes | (10, 10) |
|  | Activation | relu |
|  | Solver | sgd |
|  | Alpha | 0.0001 |
|  | Batch_size | auto |
|  | Learning_rate | 0.01 |
|  | Max_iter | 200 |
|  | Random_state | RandomState |
|  | Shuffle | True |
|  | Tol | 1.0E-4 |
|  | Learning_rate_int | 0.001 |
| Naïve Bayes | Alpha | 1 |
|  | Binarize | 0 |
|  | Fit_prior | True |
| Random forest | N_estimators | 100 |
|  | Max_depth | 10 |
|  | Min_samples_leaf | 2 |
|  | Min_samples_split | 4 |
|  | Max_features | auto |
